# Annotations

**Published:** 1894-04-07

**Authors:** 


### Annotations.

### Physiology and Property.

Mrs. Humphrey Ward, in her new novel, "Marcella," tells us that to Hallin, the peace-maker and student of Isaiah and Plato, "property meant self-realisation, and the abuse of property was no more just ground for doing away with it than the abuse of other human powers or instincts would make it reasonable to try and do away with, say, love or religion." Hallin might have gone a step farther. He might have said that since love and religion are inherent attributes in man, and the desire for property is as truly an inherent attribute, you cannot do away with either love, religion, or the desire for property; and inasmuch as the overwhelming desire for property cannot be got rid of, so neither can property itself. He who fears socialism on the one hand, and he who on the other advocates it, may cease, the one from his fears and the other from his futile labours. Physiology is a mighty illuminator, and she teaches us that so far as man's primal and inherent instincts are concerned, the case is one of "As it was in the beginning, is now, and ever shall be, world without end." In the dawn of his being man loved, worshipped, and acquired property in obedience to the mental endowments of his material protoplasm. From that time onwards he has continued to obey these imperious and universal instincts. He will still continue to obey those inhering instincts as inevitably as the needle turns to the pole, and that for the best of reasons, that he cannot help it. The socialism or collectivism which tries to do away with the possession of property by individuals may as well try to do away with the possession of heat by the sun. The only socialism which can ever become practical and concrete is the rational socialism of Christianity, of which the simple and philosophic formula is, "Whatsoever ye would that men should do unto you, do ye even so unto them."

### Professor Foster and Scientific Pin-Pointers.

Last Saturday Professor Michael Foster, of Cambridge, made an attempt to bring something of real utility out of the masses of matter in the form of papers and resolutions which have been thrown down in more or less confused heaps before the International Medical Congress at Rome. This is the age of Congresses; but the man who has listened to a few scores of papers, and stowed away a few dozens of sets of Transactions, often despairingly asks himself *Cui bono*? Professor Foster is ambitious to answer this question. "Science must be organised," he says. Scientific literature is a chaos and a futile muddle; we must bring order out of the chaos. Failing to bring order out of it, he tells us, the master who needs scientific apprentices cannot lay hands upon them; apprentices do not know where to look for masters; and the great world understands so little of science that it never knows who is an apprentice and who a master. Thus the multitudes remain ignorant, and science is brought into contempt. Professor Foster has done well to open his eyes to very obvious facts and to try to persuade other scientific men to open theirs. It is amazing how little science has done for the enlightenment of any but the few, and still more how poorly even the few are really enlightened. The bulk of men of science seem to remain in the apprenticeship stage throughout the whole of their natural lives. They master a little specialty as the Birmingham workman masters the art of putting points on pins, and there they stop. But this is not science: it is handicraft work. Not such were Newton and Faraday. Science is broad, luminous, philosophical, progressive, magnanimous. Where are the modern laboratory workers in any science of whom it can be said that their minds and their work are "broad, luminous, philosophical, progressive, magnanimous?" Ninety-nine in every hundred of them are mere "pointers of pins." Professor Michael Foster has taken a mighty task in hand, the task of making science scientific. But it is almost the task of tasks of the present day; and we urge him not to take his hand from the plough until the whole field is brought under something like worthy cultivation.

### Do Doctors Feel?

In the days of the Stuarts doctors were ridiculed; in the days of Queen Victoria they are abused. At the former period it is probable they were understood too well; to-day they are hardly understood at all. In choosing to be a doctor a man cuts himself off from a large portion of the interests of common mankind, and devotes himself almost exclusively to interests which are peculiar to himself, and concerning which the ordinary public have little knowledge and less regard. This is a distinct loss to the public, and both a loss and an injury to the medical intelligence and sentiment. Nevertheless, that we still possess the peculiar attributes of mankind we ourselves at least are fully aware. When those of us who work in the physiological laboratory, and those who profit by laboratory work, and therefore stand loyally by the laboratory workers, are called hard names and charged with the indulgence of feelings and conduct which would be horrible in a hyena or a crocodile, we feel keenly that we would like some of our traducers to possess a little of our experience, to feel a little of our conscious powerlessness in everyday affairs of life and death, and a little of our consequent longing, a longing which is almost as painful often as hunger or thirst, for more knowledge and increased power wherewith to perform the great, urgent, and distressing tasks which the world and ourselves have imposed. It may not have struck the outside world, but it is true nevertheless, that the lack of assured knowledge, and still more the lack of powerful control over diseases and sufferings, constitutes a real, serious, and lifelong medical anguish. We envy the mathematician, the engineer, the chemist, the builder, the ploughman, and all sorts and conditions of men and women whose work is such that their knowledge and power are amply adequate, not only to begin their tasks, but also to bring them to complete and desired conclusions. It is not so with us. Much of our work is still in the dark; almost all of it is uncertain in its progress and result. What can we do but cry for "light, more light," and use every possible means of obtaining it? Doctors feel deeply, not only their patients' sufferings, and their own too frequent helplessness, but also the unmerited abuse of which they are the subjects, when they try to acquire the knowledge which is power, and which is as indispensable to their own happiness as it is to the well-being of their patients.

